# Supplementary material for: Price of information in games of chance: A statistical physics approach
Source: Phys Rev Res. Author manuscript; Available in PMC 2024 Nov 30. (PMC7616869; doi:10.1103/PhysRevResearch.6.033250)
Supplement: Appendix [file EMS200757-supplement-Appendix.pdf]

APPENDIX A: OPTIMAL SELECTION OF THE BETTING STRATEGY

We consider here the problem of selecting the best betting parameter $g(\varrho)$ appearing in Eq. (11), based on the best estimate ϱ of the actual coin bias made by a player having some information I about the game.

Natural choices for $g(\varrho)$ could be $g(\varrho) = \varrho$ and $g(\varrho) = \theta(\varrho - 1/2)$, with $\theta(x)$ the Heaviside step function. In the former case $[g(\varrho) = \varrho]$, the player will choose to bet heads or tails with the same probability they have estimated for the actual bias of the coin—so if they think that the coin has a 75% bias in favor of heads, they will bet 75% of the times on head and 25% of the times on tail. In the latter case, the player will *always* bet in the direction of the bias (however strong it is), so in the example above of a coin whose bias is estimated at 75% heads, the player will always bet heads. It can be shown that this second choice leads to a better outcome on average.

Indeed, if the player's j best estimate of the pdf of the coin bias parameter is $f_I(\varrho)$, and they play according to the strategy in Eq. (11), then their probability of winning (averaged over many draws of the bias parameter) is given by

$$p_j = \int_0^1 d\varrho\, f_I(\varrho)[\varrho g(\varrho) + (1-\varrho)(1-g(\varrho))], \tag{A1}$$

where the first term in square brackets is the probability that the coin comes up heads and the player has bet heads, and the second term is the probability that the coin comes up tails and the player has bet tails. It is a trivial exercise to show that $p_j = 2/3$ if $g(\varrho) = \varrho$ and $p_j = 3/4$ if $g(\varrho) = \theta(\varrho - 1/2)$ (for the case of uniform prior $f_I(\varrho) = 1$). We can generalize this result by rewriting the integral in Eq. (A1) as follows:

$$\begin{aligned} p_j &= \int_0^1 d\varrho\, f_I(\varrho)(1-\varrho) + \int_0^1 d\varrho\, f_I(\varrho)g(\varrho)(2\varrho - 1) \\ &= 1 - \langle\varrho\rangle + \int_{1/2}^1 d\varrho\, f_I^{(j)}(\varrho)g(\varrho)(2\varrho-1) \\ &\quad - \int_0^{1/2} d\varrho\, f_I(\varrho)g(\varrho)(1-2\varrho), \end{aligned} \tag{A2}$$

where the first part of the expression is a constant, and the remaining integrals contain only non-negative terms. Now we can see that the choice $g(\varrho) = \theta(\varrho - 1/2)$ (irrespective of the amount of information I) both minimizes the negative and maximizes the positive contribution to the sum so that for *any* $g_I(\varrho)$ with support and range in [0,1] it holds that

$$\int_0^1 d\varrho\, f_I(\varrho)g(\varrho)(2\varrho-1) \leqslant \int_{1/2}^1 d\varrho\, f_I(\varrho)g(\varrho)(2\varrho-1). \tag{A3}$$

This inequality justifies the choice in Eq. (12). A completely analogous argument holds in the case where the true bias of the coin ρ is known.

APPENDIX B: CALCULATION OF $\mathbb{E}_0$ AND $\mathbb{E}_{0,\not{\rho}}$

1. Average $\mathbb{E}_0$

Assume now that no information is available to any player, but we look at the expected wealth difference of Player 2 from the point of view of an omniscient observer, who knows that the coin tosses are indeed independent and biased, with bias ρ. Using the identity $\frac{1}{x} = \int_0^\infty ds\, e^{-sx}$, for $x > 0$ to lift the denominator N_t, we can write (exploiting sum factorization)

$$\begin{aligned} \mathbb{E}_0\left[\frac{\delta_{\sigma_t,\sigma_t^{(2)}}}{N_t}\right] &= \int_0^\infty ds \int_0^1 d\rho_1\, f_0(\rho_1)\cdots\int_0^1 d\rho_N\, f_0(\rho_N) \sum_{\sigma_t=\pm1} P_\rho(\sigma_t) \sum_{\sigma_t^{(1)}=\pm1} \tilde{P}_{\rho_1}\big(\sigma_t^{(1)}\big) \\ &\quad \times e^{-s\delta_{\sigma_t,\sigma_t^{(1)}}} \sum_{\sigma_t^{(2)}} \tilde{P}_{\rho_2}\big(\sigma_t^{(2)}\big)\delta_{\sigma_t,\sigma_t^{(2)}} e^{-s\delta_{\sigma_t,\sigma_t^{(2)}}} \cdots \sum_{\sigma_t^{(N)}} \tilde{P}_{\rho_N}\big(\sigma_t^{(N)}\big) e^{-s\delta_{\sigma_t,\sigma_t^{(N)}}} \end{aligned} \tag{B1}$$

$$= \int_0^\infty ds \int_0^1 d\rho_2\, f_0(\rho_2)[\rho g(\rho_2) I_+(N-1,s) + (1-\rho)(1-g(\rho_2))I_-(N-1,s)], \tag{B2}$$

where

$$I_+(N-1,s) = e^{-s}\prod_{k\neq2}^N\left[\int_0^1 d\varrho\, f_0(\varrho)\sum_{\sigma=\pm1}\tilde{P}_\varrho(\sigma)\exp(-s\delta_{\sigma,+1})\right] = e^{-s}\prod_{k\neq2}^N\left[\int_0^1 d\varrho\, f_0(\varrho)[g(\varrho)e^{-s} + (1-g(\varrho))]\right], \tag{B3}$$

and

$$I_-(N-1,s) = e^{-s}\prod_{k\neq2}^N\left[\int_0^1 d\varrho\, f_0(\varrho)\sum_{\sigma=\pm1}\tilde{P}_\varrho(\sigma)\exp(-s\delta_{\sigma,-1})\right] = e^{-s}\prod_{k\neq2}^N\left[\int_0^1 d\varrho\, f_0(\varrho)[g(\varrho) + (1-g(\varrho))e^{-s}]\right]. \tag{B4}$$

Using $f_0(\rho_2) = 1$, $g(\rho_2) = \theta(\rho_2 - 1/2)$ and the symmetry condition $1 - g(\rho_2) = g(1 - \rho_2)$ (which further implies $\int_0^1 d\rho_2\, g(\rho_2) = \int_0^1 d\rho_2 (1 - g(\rho_2))$), we get that the ρ dependence drops out, and

$$\mathbb{E}_0\left[\frac{\delta_{\sigma_t,\sigma_t^{(2)}}}{N_t}\right] = \int_0^\infty ds\, e^{-s}\left(\frac{1}{2}e^{-s} + \frac{1}{2}\right)^{N-1}\int_0^1 d\rho_2 g(\rho_2) = \frac{2-2^{1-N}}{N}\int_0^1 d\rho_2 g(\rho_2), \tag{B5}$$

independent of ρ. Computing the integral explicitly, leads to Eq. (22), showing that the expectation is always negative. This is expected as—in the event of all players betting on the wrong outcome—the "dealer" would keep the total entry fee collected from the players, which would not be redistributed among them.

In the next subsection, we perform the same calculation but this time assuming the viewpoint of player 2, who does not know the actual value of the coin bias ρ and will therefore use their own estimate ρ_2 in lieu of ρ.

2. Average $\mathbb{E}_{0,\not\rho}$

Here we compute the expected wealth change, from the point of view of player 2 (or, equivalently, any other uninformed player), using the estimate ρ_2 to replace the true, unknown, coin bias ρ. We refer to this expectation as $\mathbb{E}_{0,\not\rho}[\Delta W_2]$ to remark the use of a proxy for ρ in the computation of Eq. (20), rather than leaving it as a free parameter, even though it eventually drops out. The computation is carried out as before, starting from

$$\mathbb{E}_{0,\not\rho}\left[\frac{\delta_{\sigma_t,\sigma_t^{(2)}}}{N_t}\right] = \int_0^\infty ds \int_0^1 d\rho_1\, f_0(\rho_1)\cdots\int_0^1 d\rho_N\, f_0(\rho_N)\sum_{\sigma_t=\pm1}P_{\rho_2}(\sigma_t)\sum_{\sigma_t^{(1)}=\pm1}\tilde{P}_{\rho_1}\big(\sigma_t^{(1)}\big)$$
$$\times\, e^{-s\delta_{\sigma_t,\sigma_t^{(1)}}}\sum_{\sigma_t^{(2)}}\tilde{P}_{\rho_2}\big(\sigma_t^{(2)}\big)\delta_{\sigma_t,\sigma_t^{(2)}}e^{-s\delta_{\sigma_t,\sigma_t^{(2)}}}\cdots\sum_{\sigma_t^{(N)}}\tilde{P}_{\rho_N}\big(\sigma_t^{(N)}\big)e^{-s\delta_{\sigma_t,\sigma_t^{(N)}}} \tag{B6}$$

$$= \int_0^\infty ds\int_0^1 d\rho_2\, f_0(\rho_2)[\rho_2 g(\rho_2) I_+(N-1,s) + (1-\rho_2)(1-g(\rho_2))I_-(N-1,s)], \tag{B7}$$

where $I_\pm(N-1,s)$ are defined in Eqs. (B3) and (B4).

Assuming again that all uninformed players will estimate the bias parameter of the coin uniformly in [0,1], we get again for $g(\varrho) = \theta(\varrho - 1/2)$ that

$$I_+(N-1,s) = I_-(N-1,s) = e^{-s}\left[\frac{1}{2}e^{-s} + \frac{1}{2}\right]^{N-1}, \tag{B8}$$

from which it follows that

$$\mathbb{E}_{0,\not\rho}\left[\frac{\delta_{\sigma_t,\sigma_t^{(2)}}}{N_t}\right] = \int_0^\infty ds\, e^{-s}\left(\frac{1}{2}e^{-s} + \frac{1}{2}\right)^{N-1}$$
$$\times\, \mathbb{E}_{0,\not\rho}\big[\delta_{\sigma_t,\sigma_t^{(2)}}\big] = \frac{3}{4}\frac{2-2^{1-N}}{N}, \tag{B9}$$

where

$$\mathbb{E}_{0,\not\rho}\big[\delta_{\sigma_t,\sigma_t^{(2)}}\big] = \int_0^1 d\rho_2(\rho_2 g(\rho_2) + (1-\rho_2)(1-g(\rho_2)))$$
$$= \frac{3}{4} \tag{B10}$$

is the winning probability estimated by player 2, who does not have any knowledge of the actual bias of the coin.

Taking the expectation of Eq. (6) and inserting Eq. (B9), we get

$$\mathbb{E}_{0,\not\rho}[\Delta W_2] = -M + M\frac{3(1-2^{-N})}{2} = \frac{1}{2}M\left(1 - \frac{3}{2^N}\right). \tag{B11}$$

Comparing Eqs. (B11) and (22), we find that $\mathbb{E}_{0,\not\rho}[\Delta W_2] > \mathbb{E}_0[\Delta W_2]$. This is intuitive, as in $\mathbb{E}_{0,\not\rho}$ the player is using the same parameter ρ_2 to estimate their best strategy *and* the actual bias of the coin, which obviously leads to a more optimistic outlook on their game.

APPENDIX C: CALCULATION OF WINNING PROBABILITY FOR THE DATA HOLDER AND ASYMPTOTICS FOR $R \to \infty$

We compute here the probability that the data holder wins the bet in a single round (estimated by the data holder themselves) assuming that they hold a string of the past R outcomes and will use it to place their bet

$$\mathbb{E}_R\big[\delta_{\sigma_t,\sigma_t^{(1)}}\big] = \int_0^1 d\rho_1\, f_R(\rho_1)\int_0^1 d\rho_2\, f_0(\rho_2)\cdots\int_0^1 d\rho_N\, f_0(\rho_N)\sum_{\sigma_t=\pm1}P_{\rho_1}(\sigma_t)\sum_{\sigma_t^{(1)}=\pm1}\tilde{P}_{\rho_1}\big(\sigma_t^{(1)}\big)$$
$$\times\, \delta_{\sigma_t,\sigma_t^{(1)}}\sum_{\sigma_t^{(2)}}\tilde{P}_{\rho_2}\big(\sigma_t^{(2)}\big)\cdots\sum_{\sigma_t^{(N)}}\tilde{P}_{\rho_N}\big(\sigma_t^{(N)}\big) = \int_0^1 d\rho_1\, f_R(\rho_1)[\rho_1 g(\rho_1) + (1-\rho_1)(1-g(\rho_1))]. \tag{C1}$$

Here, we have used the fact that the data holder (player 1) will estimate the actual coin bias *and* the parameter appearing in their own best strategy as ρ_1, which is drawn from the posterior pdf $f_R(\rho_1)$ given in Eq. (25). We also used that the betting distributions $\tilde{P}_\varrho$ are normalized to unity.

Performing the elementary integral in Eq. (C1) with $g(\rho_1) = \theta(\rho_1 - 1/2)$ and $f_R(\rho_1)$ given in Eq. (25) we get

$$\mathbb{E}_R[\delta_{\sigma_t,\sigma_t^{(1)}}] = \frac{(R+1)!}{H!(R-H)!2^{R+2}} \times [F(H+1, R-H) + F(R-H+1, H)] =: \Xi_R(H), \quad \text{(C2)}$$

where $F(x, y)$ is defined in Eq. (29) in terms of the ${}_2F_1$ hypergeometric function.

We can now compute the asymptotics $\Xi_R(\alpha R)$ for large R for a fraction $0 \leqslant \alpha \leqslant 1$ of heads seen in the long string of data held by player 1. From Eq. (C2), we need the following saddle-point asymptotics:

$$F(\alpha R + 1, R - \alpha R) = \int_0^1 dt (1+t)^{\alpha R+1}(1-t)^{R(1-\alpha)} = \int_0^1 dt(1+t)\exp[R g_\alpha(t)] \quad \text{(C3)}$$

$$F(R - \alpha R + 1, \alpha R) = \int_0^1 dt (1+t)^{R-\alpha R+1}(1-t)^{\alpha R} = \int_0^1 dt(1+t)\exp[R g_{1-\alpha}(t)], \quad \text{(C4)}$$

with

$$g_\alpha(t) = \alpha \log(1+t) + (1-\alpha)\log(1-t), \quad \text{(C5)}$$

from which

$$g'_\alpha(t^\star) = 0 \Rightarrow t^\star = 2\alpha - 1, \quad \text{(C6)}$$

which is within the integration interval for $1/2 < \alpha < 1$. Using the Stirling approximation for the prefactor

$$\frac{(R+1)!}{H!(R-H)!2^{R+m}} \sim e^{-R[\alpha\log(1-\alpha)+(1-\alpha)\log(1-\alpha)+\log(2)]} \times \frac{1}{2^m}\sqrt{\frac{R}{2\pi\alpha(1-\alpha)}} \quad \text{(C7)}$$

and computing $g_\alpha(t^\star)$, we see that the leading exponential terms cancel out exactly, and for $m = 2$ combining all the prefactors together we finally obtain Eq. (31) of the main text.

APPENDIX D: CALCULATION OF $\mathbb{E}_{R,r}$

We start from Eq. (36)

$$\mathbb{E}_{R,r}\left[\frac{\delta_{\sigma_t,\sigma_t^{(1)}}}{N_t}\right] = \int_0^\infty ds \int_0^1 d\rho_1 f_{R,r}(\rho_1) \int_0^1 d\rho_2 f_{r,r}(\rho_2)\cdots\int_0^1 d\rho_N f_0(\rho_N) \sum_{\sigma_t=\pm1} P_{\rho_1}(\sigma_t) \sum_{\sigma_t^{(1)}=\pm1} \tilde{P}_{\rho_1}(\sigma_t^{(1)}) \times \delta_{\sigma_t,\sigma_t^{(1)}} e^{-s\delta_{\sigma_t,\sigma_t^{(1)}}} \sum_{\sigma_t^{(2)}} \tilde{P}_{\rho_2}(\sigma_t^{(2)}) e^{-s\delta_{\sigma_t,\sigma_t^{(2)}}} \cdots \sum_{\sigma_t^{(N)}} \tilde{P}_{\rho_N}(\sigma_t^{(N)}) e^{-s\delta_{\sigma_t,\sigma_t^{(N)}}} \quad \text{(D1)}$$

$$= \int_0^\infty ds \int_0^1 d\rho_1 f_{R,r}(\rho_1) \int_0^1 d\rho_2 f_{r,r}(\rho_2)[\rho_1 g(\rho_1) I_+(N-2, s)\chi_+(\rho_2, s) + (1-\rho_1)(1-g(\rho_1))I_-(N-2, s)\chi_-(\rho_2, s)], \quad \text{(D2)}$$

where $I_\pm$ are defined in Eqs. (B3) and (B4) and

$$\chi_+(\varrho, s) = g(\varrho)e^{-s} + 1 - g(\varrho), \quad \text{(D3)}$$

$$\chi_-(\varrho, s) = g(\varrho) + (1 - g(\varrho))e^{-s}. \quad \text{(D4)}$$

Here, we have used factorization of the summations after N_t is lifted up using the s-identity, and the fact that $N - 2$ summations (corresponding to the uninformed players) are identical.

Also, for uniform priors f_0, we have that

$$I_+(N-2, s) = I_-(N-2, s) = e^{-s}\left[\frac{1}{2}e^{-s} + \frac{1}{2}\right]^{N-2}, \quad \text{(D5)}$$

leading to

$$\mathbb{E}_{R,r}\left[\frac{\delta_{\sigma_t,\sigma_t^{(1)}}}{N_t}\right] = C_N X_{R,r}(x, h) + D_N Y_{R,r}(x, h), \quad \text{(D6)}$$

where

$$C_N = \int_0^\infty ds\, e^{-2s}\left[\frac{1}{2}e^{-s} + \frac{1}{2}\right]^{N-2} = \frac{2N + 2^{2-N} - 4}{(N-1)N}, \quad \text{(D7)}$$

$$D_N = \int_0^\infty ds\, e^{-s}\left[\frac{1}{2}e^{-s} + \frac{1}{2}\right]^{N-2} = \frac{2 - 2^{2-N}}{N-1}, \quad \text{(D8)}$$

and

$$X_{R,r}(x, h) = \int_0^1 d\rho_1 f_{R,r}(\rho_1) \int_0^1 d\rho_2 f_{r,r}(\rho_2)[\rho_1 g(\rho_1) g(\rho_2) + (1-\rho_1)(1-g(\rho_1))(1-g(\rho_2))], \quad \text{(D9)}$$

$$Y_{R,r}(x, h) = \int_0^1 d\rho_1 f_{R,r}(\rho_1) \int_0^1 d\rho_2 f_{r,r}(\rho_2)[\rho_1 g(\rho_1) \times (1-g(\rho_2)) + (1-\rho_1)(1-g(\rho_1))g(\rho_2)]. \quad \text{(D10)}$$

We start from

$$X_{R,r}(x,h)=\int_0^1 d\rho_1 f_{R,r}(\rho_1)\int_0^1 d\rho_2 f_{r,r}(\rho_2)\times[\rho_1 g(\rho_2)g(\rho_1)+(1-\rho_1)(1-g(\rho_2))\times(1-g(\rho_1))]=J_1+J_2, \tag{D11}$$

where

$$J_1=\int_{1/2}^1 d\rho_2 f_{r,r}(\rho_2)\int_{1/2}^1 d\rho_1 f_{R,r}(\rho_1)\rho_1, \tag{D12}$$

$$J_2=\int_0^{1/2} d\rho_2 f_{r,r}(\rho_2)\int_0^{1/2} d\rho_1 f_{R,r}(\rho_1)(1-\rho_1). \tag{D13}$$

First, we have

$$\int_{1/2}^1 d\rho_2 f_{r,r}(\rho_2)=C_{r,h}\frac{1}{2^{r+1}}F(h,r-h), \tag{D14}$$

$$\int_{1/2}^1 d\rho_1 f_{R,r}(\rho_1)\rho_1=\frac{C_{R,x+h}}{2^{R+2}}F(h+x+1,R-(h+x)), \tag{D15}$$

$$\int_0^{1/2} d\rho_2 f_{r,r}(\rho_2)=C_{r,h}\frac{1}{2^{r+1}}F(r-h,h), \tag{D16}$$

$$\int_0^{1/2} d\rho_1 f_{R,r}(\rho_1)(1-\rho_1)=\frac{C_{R,h+x}}{2^{R+2}}F(R-(h+x)+1,h+x), \tag{D17}$$

where $C_{r,h}=(r+1)!/(h!(r-h)!)$ and $C_{R,x+h}=(R+1)!/((h+x)!(R-(h+x))!)$. $F(x,y)$ is defined in Eq. (29).

Therefore,

$$X_{R,r}(x,h)=\frac{C_{R,h+x}C_{r,h}}{2^{R+r+3}}\times\{F(h,r-h)F(h+x+1,R-(h+x))+F(r-h,h)F(R-(h+x)+1,h+x)\}. \tag{D18}$$

Similarly,

$$Y_{R,r}(x,h)=\int_0^1 d\rho_1 f_{R,r}(\rho_1)\int_0^1 d\rho_2 f_{r,r}(\rho_2)[\rho_1 g(\rho_1)\times(1-g(\rho_2))+(1-\rho_1)(1-g(\rho_1))g(\rho_2)]=K_1+K_2, \tag{D19}$$

where

$$K_1=\int_0^{1/2} d\rho_2 f_{r,r}(\rho_2)\int_{1/2}^1 d\rho_1 f_{R,r}(\rho_1)\rho_1, \tag{D20}$$

$$K_2=\int_{1/2}^1 d\rho_2 f_{r,r}(\rho_2)\int_0^{1/2} d\rho_1 f_{R,r}(\rho_1)(1-\rho_1). \tag{D21}$$

Using the previously computed elementary integrals, we can immediately write

$$Y_{R,r}(x,h)=\frac{C_{R,h+x}C_{r,h}}{2^{R+r+3}}\times\{F(r-h,h)F(h+x+1,R-(h+x))+F(h,r-h)F(R-(h+x)+1,h+x)\}. \tag{D22}$$

APPENDIX E: CALCULATION OF $\mathbb{E}_{r,r}$

We start from Eq. (E2)

$$\mathbb{E}_{r,r}\left[\frac{\delta_{\sigma_t,\sigma_t^{(2)}}}{N_t}\right]=\int_0^\infty ds\int_0^1 d\rho_1\, f_{r,r}(\rho_1)\int_0^1 d\rho_2\, f_{r,r}(\rho_2)\cdots\int_0^1 d\rho_N\, f_0(\rho_N)\sum_{\sigma_t=\pm1}P_{\rho_2}(\sigma_t)\sum_{\sigma_t^{(2)}=\pm1}\tilde{P}_{\rho_2}(\sigma_t^{(2)})\times\delta_{\sigma_t,\sigma_t^{(2)}}e^{-s\delta_{\sigma_t,\sigma_t^{(2)}}}\sum_{\sigma_t^{(1)}}\tilde{P}_{\rho_1}(\sigma_t^{(1)})e^{-s\delta_{\sigma_t,\sigma_t^{(1)}}}\cdots\sum_{\sigma_t^{(N)}}\tilde{P}_{\rho_N}(\sigma_t^{(N)})e^{-s\delta_{\sigma_t,\sigma_t^{(N)}}} \tag{E1}$$

$$=\int_0^\infty ds\int_0^1 d\rho_1\, f_{r,r}(\rho_1)\int_0^1 d\rho_2\, f_{r,r}(\rho_2)[\rho_2 g(\rho_2)I_+(N-2,s)\chi_+(\rho_1,s)+(1-\rho_2)(1-g(\rho_2))I_-(N-2,s)\chi_-(\rho_1,s)], \tag{E2}$$

where $\chi_\pm$ is defined in Eqs. (D3) and (D4), and $I_\pm$ are defined in Eqs. (B3) and (B4). Also, using Eq. (D5), we have that

$$\mathbb{E}_{r,r}\left[\frac{\delta_{\sigma_t,\sigma_t^{(2)}}}{N_t}\right]=C_N X_r(h)+D_N Y_r(h), \tag{E3}$$

where C_N and D_N are defined, respectively, in Eqs. (D7) and (D8), also

$$X_r(h)=\int_0^1 d\rho_1 f_{r,r}(\rho_1)\int_0^1 d\rho_2 f_{r,r}(\rho_2)[\rho_2 g(\rho_2)g(\rho_1)+(1-\rho_2)(1-g(\rho_2))(1-g(\rho_1))], \tag{E4}$$

$$Y_r(h)=\int_0^1 d\rho_1 f_{r,r}(\rho_1)\int_0^1 d\rho_2 f_{r,r}(\rho_2)[\rho_2 g(\rho_2)(1-g(\rho_1))+(1-\rho_2)(1-g(\rho_2))g(\rho_1)]. \tag{E5}$$

We start from

$$X_r(h) = \int_0^1 d\rho_1 f_{r,r}(\rho_1) \int_0^1 d\rho_2 f_{r,r}(\rho_2)[\rho_2 g(\rho_2)g(\rho_1) + (1-\rho_2)(1-g(\rho_2))(1-g(\rho_1))] = J_1' + J_2', \tag{E6}$$

where

$$J_1' = \int_{1/2}^1 d\rho_1 f_{r,r}(\rho_1) \int_{1/2}^1 d\rho_2 f_{r,r}(\rho_2)\rho_2, \tag{E7}$$

$$J_2' = \int_0^{1/2} d\rho_1 f_{r,r}(\rho_1) \int_0^{1/2} d\rho_2 f_{r,r}(\rho_2)(1-\rho_2). \tag{E8}$$

First, we have

$$\int_{1/2}^1 d\rho_1 f_{r,r}(\rho_1) = C_{r,h}\frac{1}{2^{r+1}}F(h, r-h), \tag{E9}$$

$$\int_{1/2}^1 d\rho_2 f_{r,r}(\rho_2)\rho_2 = C_{r,h}\frac{1}{2^{r+2}}F(h+1, r-h), \tag{E10}$$

$$\int_0^{1/2} d\rho_1 f_{r,r}(\rho_1) = C_{r,h}\frac{1}{2^{r+1}}F(r-h, h), \tag{E11}$$

$$\int_0^{1/2} d\rho_2 f_{r,r}(\rho_2)(1-\rho_2) = C_{r,h}\frac{1}{2^{r+2}}F(r-h+1, h), \tag{E12}$$

where $C_{r,h} = (r+1)!/(h!(r-h)!)$ and $F(x, y)$ is defined in Eq. (29).

Therefore,

$$X_r(h) = (C_{r,h})^2 \frac{1}{2^{2r+3}}\{F(h, r-h)F(h+1, r-h) + F(r-h, h)F(r-h+1, h)\}. \tag{E13}$$

Similarly,

$$Y_r(h) = \int_0^1 d\rho_1 f_{r,r}(\rho_1) \int_0^1 d\rho_2 f_{r,r}(\rho_2) \times [\rho_2 g(\rho_2)(1-g(\rho_1)) + (1-\rho_2)(1-g(\rho_2))g(\rho_1)] = K_1' + K_2', \tag{E14}$$

where

$$K_1' = \int_0^{1/2} d\rho_1 f_{r,r}(\rho_1) \int_{1/2}^1 d\rho_2 f_{r,r}(\rho_2)\rho_2, \tag{E15}$$

$$K_2' = \int_{1/2}^1 d\rho_1 f_{r,r}(\rho_1) \int_0^{1/2} d\rho_2 f_{r,r}(\rho_2)(1-\rho_2). \tag{E16}$$

Using the previously computed elementary integrals, we can immediately write

$$Y_r(h) = (C_{r,h})^2 \frac{1}{2^{2r+3}}\{F(r-h, h)F(h+1, r-h) + F(h, r-h)F(r-h+1, h)\}. \tag{E17}$$

APPENDIX F: EXPLICIT EXPRESSIONS FOR $\Psi_{\min}$ AND $\Psi_{\max}$ FOR $M = 1$

Let us put ourselves in the simplified setting $M = 1$ (single round of the game). Recalling Eq. (6) and the various intermediate results,

$$\mathbb{E}_R\left[\frac{\delta_{\sigma_t,\sigma_t^{(1)}}}{N_t}\right] = \frac{2-2^{1-N}}{N}\Xi_R(H), \tag{F1}$$

$$\mathbb{E}_{R,r}\left[\frac{\delta_{\sigma_t,\sigma_t^{(1)}}}{N_t}\right] = C_N X_{R,r}(x, h) + D_N Y_{R,r}(x, h), \tag{F2}$$

$$\mathbb{E}_{r,r}\left[\frac{\delta_{\sigma_t,\sigma_t^{(2)}}}{N_t}\right] = C_N X_r(h) + D_N Y_r(h), \tag{F3}$$

$$\mathbb{E}_0\left[\frac{\delta_{\sigma_t,\sigma_t^{(2)}}}{N_t}\right] = \frac{1}{2}\frac{2-2^{1-N}}{N}, \tag{F4}$$

we get

$$\Psi_{\min}/\phi = \max[(2-2^{1-N})\Xi_R(h+x) - NC_N X_{R,r}(x, h) - ND_N Y_{R,r}(x, h), 0], \tag{F5}$$

$$\Psi_{\max}/\phi = \max[NC_N X_r(h) + ND_N Y_r(h) - \tfrac{1}{2}(2-2^{1-N}), 0], \tag{F6}$$

where

$$\Xi_R(H) = \frac{(R+1)!}{H!(R-H)!2^{R+2}}[F(H+1, R-H) + F(R-H+1, H)], \tag{F7}$$

with

$$F(x, y) = \int_0^1 dt\,(1+t)^x(1-t)^y = \frac{{}_2F_1(1, -x; y+2; -1)}{y+1}. \tag{F8}$$

Furthermore, we have

$$C_N = \frac{2N + 2^{2-N} - 4}{(N-1)N}, \tag{F9}$$

$$D_N = \frac{2-2^{2-N}}{N-1}, \tag{F10}$$

$$X_r(h) = (C_{r,h})^2 \frac{1}{2^{2r+3}}\{F(h, r-h)F(h+1, r-h) + F(r-h, h)F(r-h+1, h)\}, \tag{F11}$$

$$Y_r(h) = (C_{r,h})^2 \frac{1}{2^{2r+3}}\{F(r-h, h)F(h+1, r-h) + F(h, r-h)F(r-h+1, h)\}, \tag{F12}$$

$$X_{R,r}(x,h) = \frac{C_{R,h+x}C_{r,h}}{2^{R+r+3}} \times \{F(h, r-h)F(h+x+1, R-(h+x)) + F(r-h, h)F(R-(h+x)+1, h+x)\}, \tag{F13}$$

$$Y_{R,r}(x,h) = \frac{C_{R,h+x}C_{r,h}}{2^{R+r+3}} \times \{F(r-h, h)F(h+x+1, R-(h+x)) + F(h, r-h)F(R-(h+x) + 1, h+x)\}, \tag{F14}$$

in terms of constants $C_{p,q} = (p+1)!/(q!(p-q)!)$.
